# Supplementary material for: Identification of Reference Genes for RT-qPCR Analysis in Gleditsia microphylla under Abiotic Stress and Hormone Treatment
Source: Genes (Basel). 2022 Jul 10;13(7):1227. doi: 10.3390/genes13071227 (PMC9315665; doi:10.3390/genes13071227)
Supplement: Supplementary file 1 [file genes-13-01227-s001.zip › Figure S1 Melting curves for the candidate reference genes and target genes.pdf]

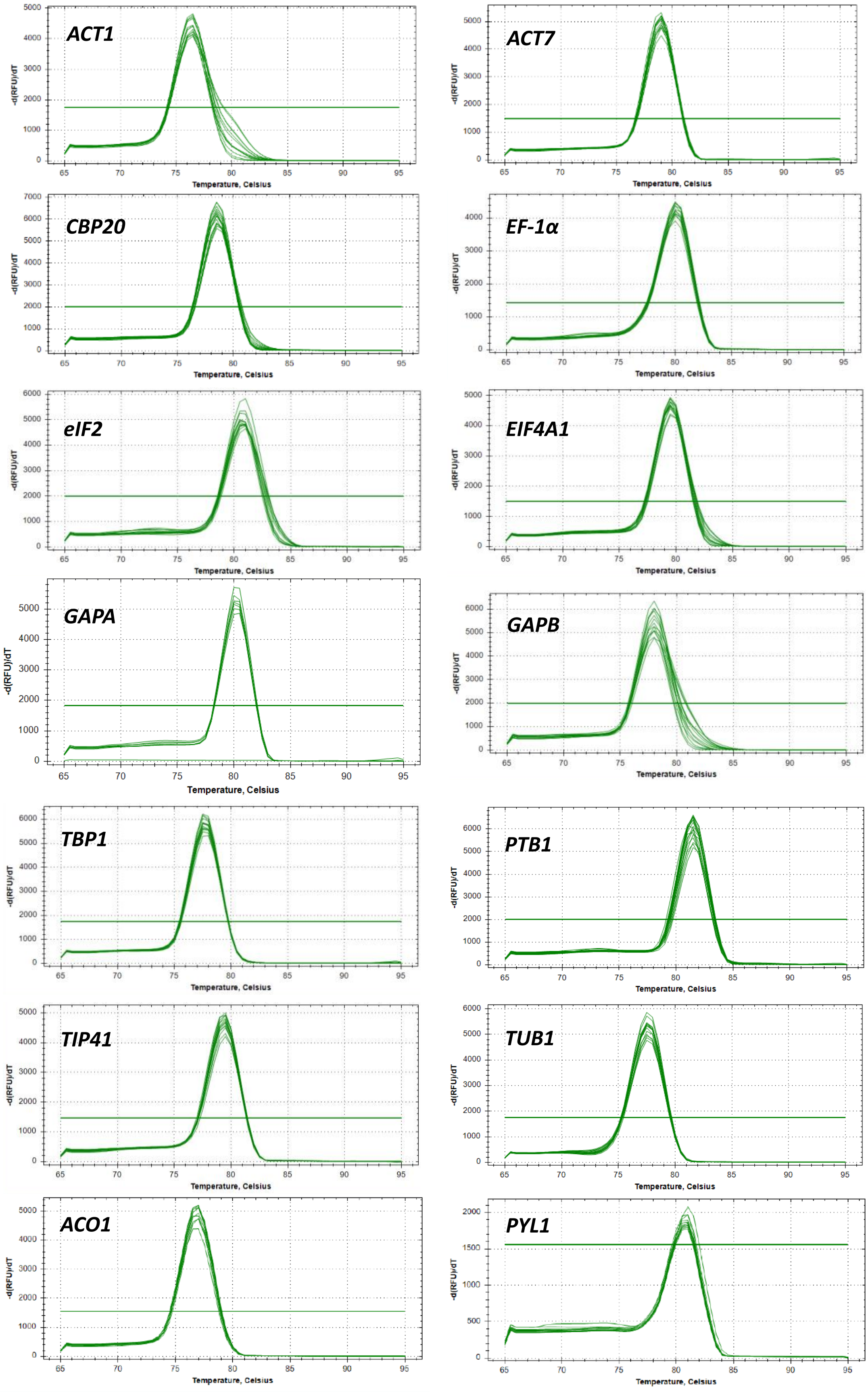
ACT1
ACT7
CBP20
EF-1α
eIF2
EIF4A1
GAPA
GAPB
TBP1
PTB1
TIP41
TUB1
ACO1
PYL1
-d(RFU)/dT
Temperature, Celsius

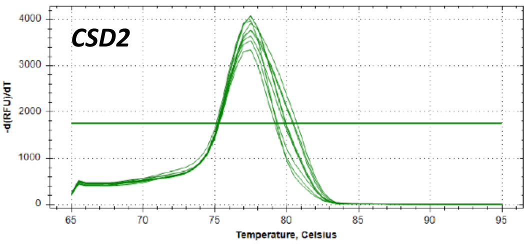

Figure S1. Melting curves for the candidate reference genes and target genes. Melting temperatures were visualized by plotting the negative first derivative of fluorescence relative to the temperature in Celsius (–(d/dT)). Melting curves of twelve candidate reference genes and three target genes show a simple peak.
