# Supplementary material for: Identification of Reference Genes for RT-qPCR Analysis in Gleditsia microphylla under Abiotic Stress and Hormone Treatment
Source: Genes (Basel). 2022 Jul 10;13(7):1227. doi: 10.3390/genes13071227 (PMC9315665; doi:10.3390/genes13071227)
Supplement: Supplementary file 1 [file genes-13-01227-s001.zip › Figure S2 Standard curves of the candidate reference genes and target genes.pdf]

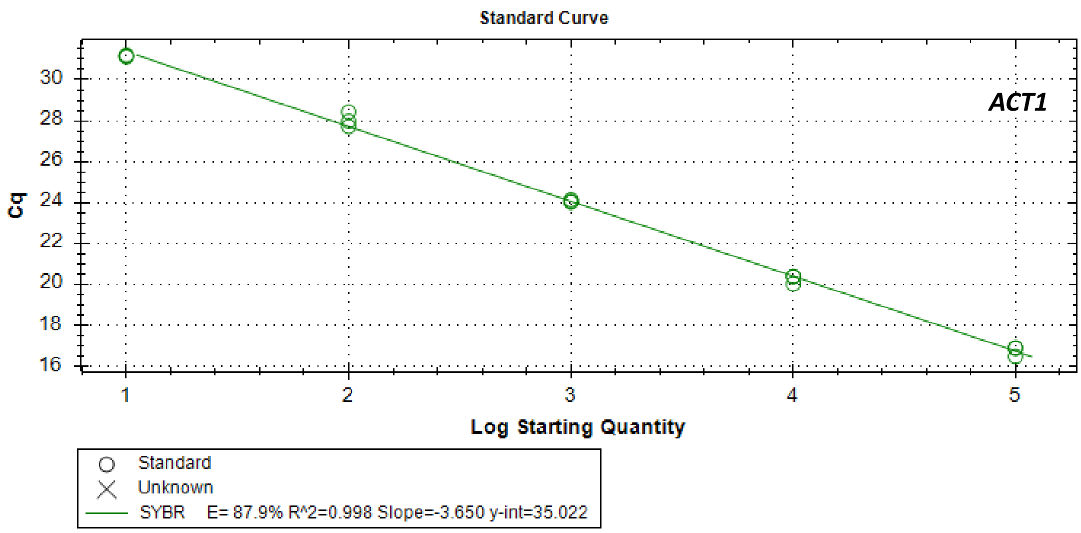
Standard Curve
ACT1
Cq
30
28
26
24
22
20
18
16
1
2
3
4
5
Log Starting Quantity
Standard
Unknown
SYBR E= 87.9% R^2=0.998 Slope=-3.650 y-int=35.022

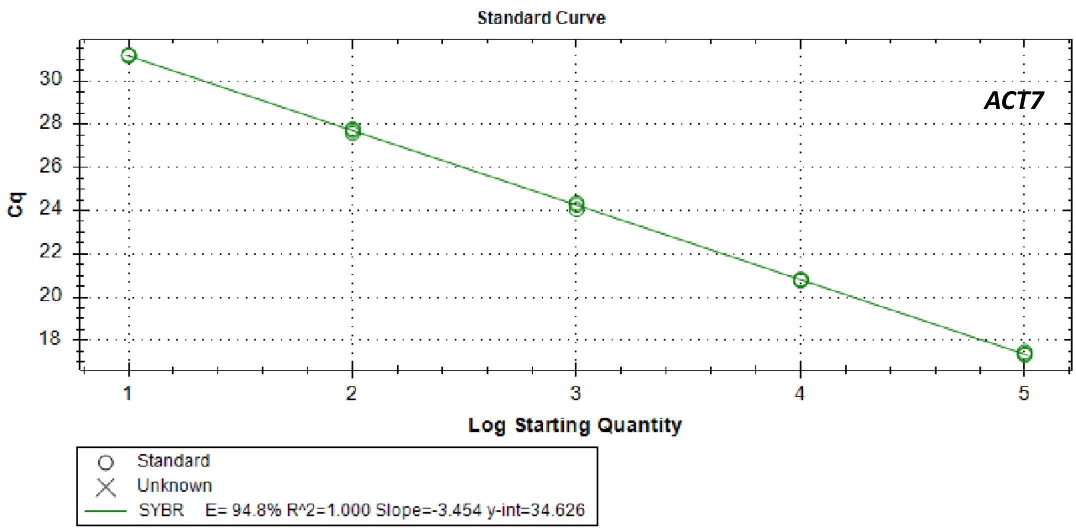
Standard Curve
ACT7
Cq
30
28
26
24
22
20
18
1
2
3
4
5
Log Starting Quantity
Standard
Unknown
SYBR E= 94.8% R^2=1.000 Slope=-3.454 y-int=34.626

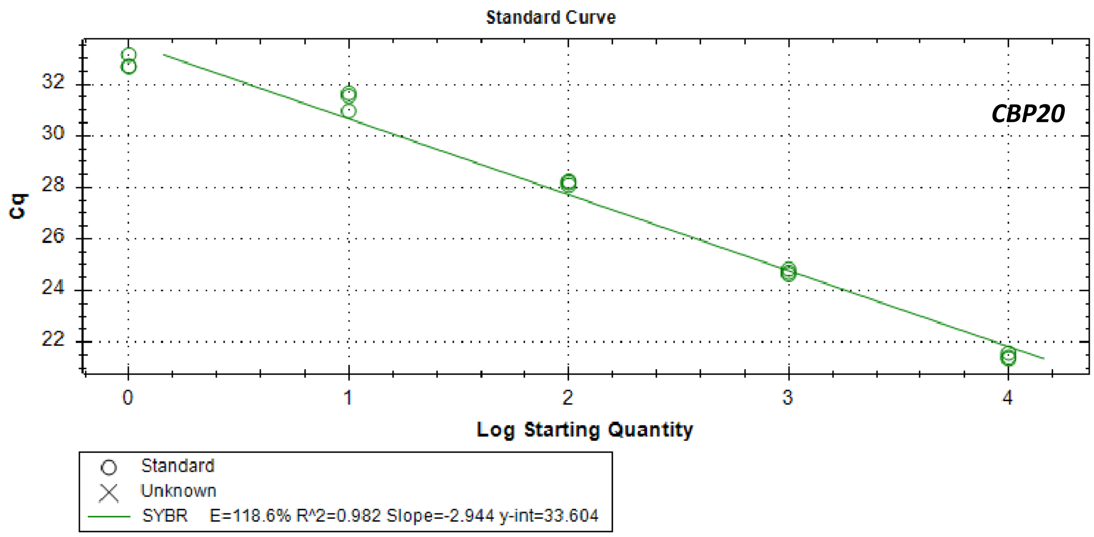
Standard Curve
CBP20
Cq
32
30
28
26
24
22
0
1
2
3
4
Log Starting Quantity
Standard
Unknown
SYBR E=118.6% R^2=0.982 Slope=-2.944 y-int=33.604

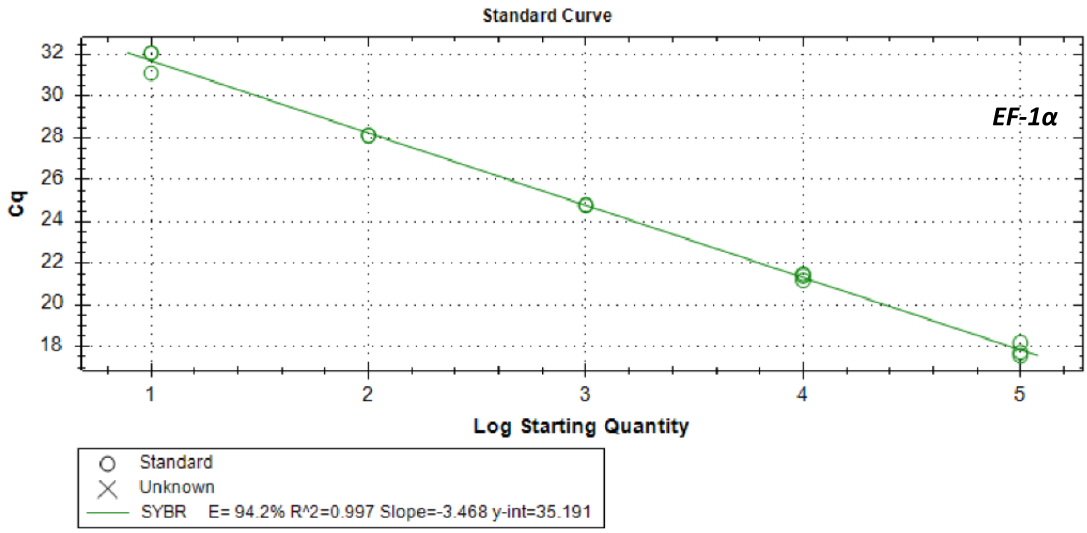
Standard Curve
EF-1α
Cq
32
30
28
26
24
22
20
18
1
2
3
4
5
Log Starting Quantity
Standard
Unknown
SYBR E= 94.2% R^2=0.997 Slope=-3.468 y-int=35.191

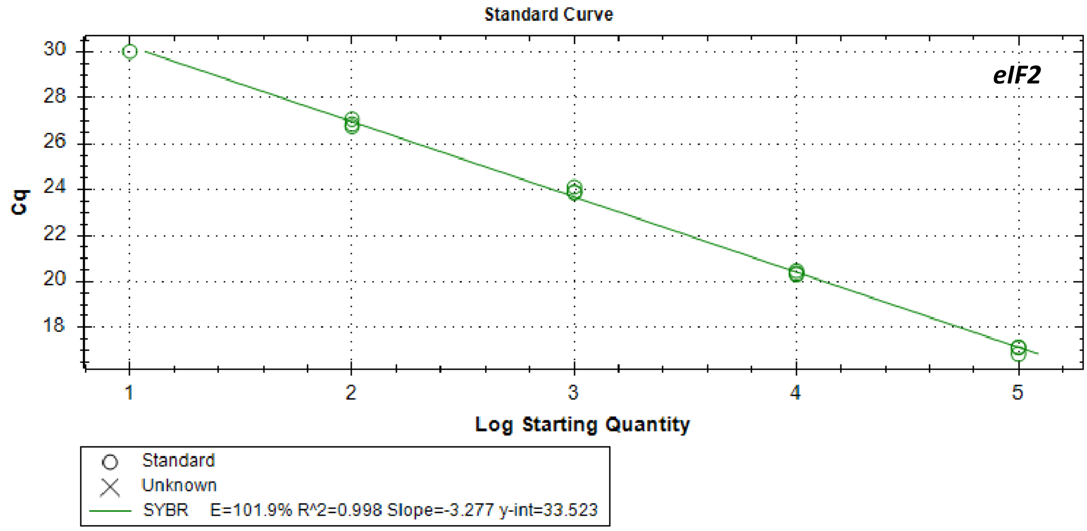
Standard Curve
eIF2
Cq
30
28
26
24
22
20
18
1
2
3
4
5
Log Starting Quantity
Standard
Unknown
SYBR E=101.9% R^2=0.998 Slope=-3.277 y-int=33.523

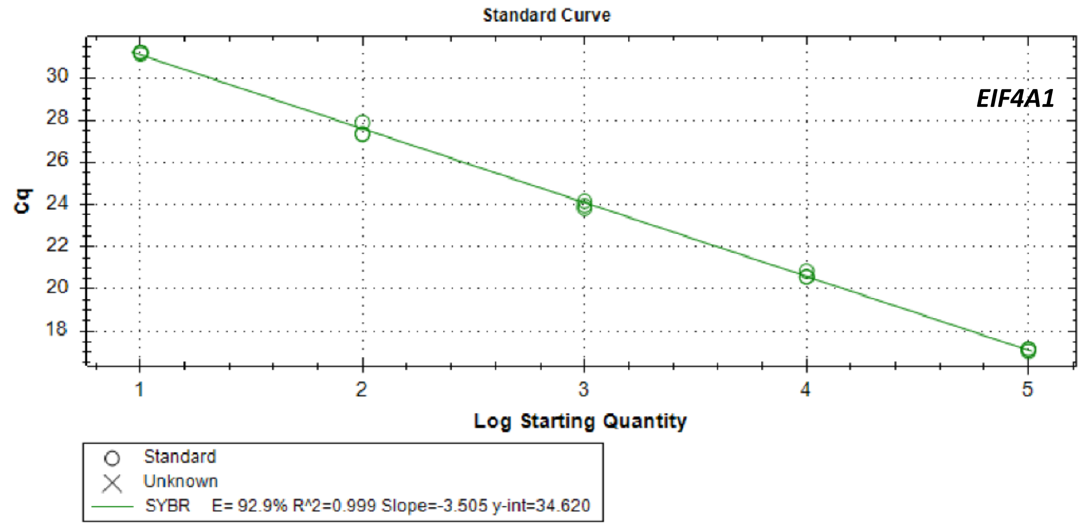
Standard Curve
EIF4A1
Cq
30
28
26
24
22
20
18
1
2
3
4
5
Log Starting Quantity
Standard
Unknown
SYBR E= 92.9% R^2=0.999 Slope=-3.505 y-int=34.620

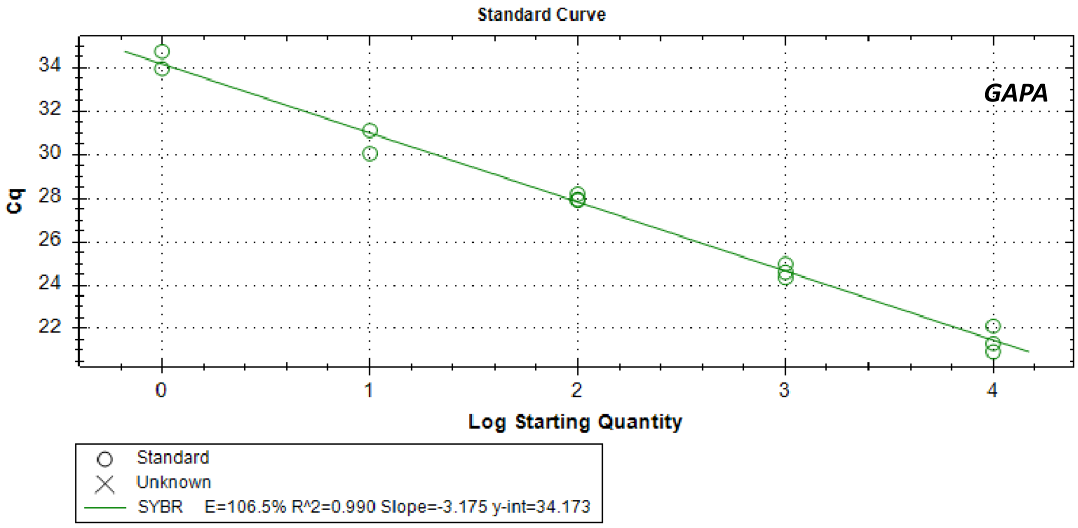
Standard Curve
GAPA
34
32
30
28
26
24
22
Cq
0
1
2
3
4
Log Starting Quantity
Standard
Unknown
SYBR E=106.5% R^2=0.990 Slope=-3.175 y-int=34.173

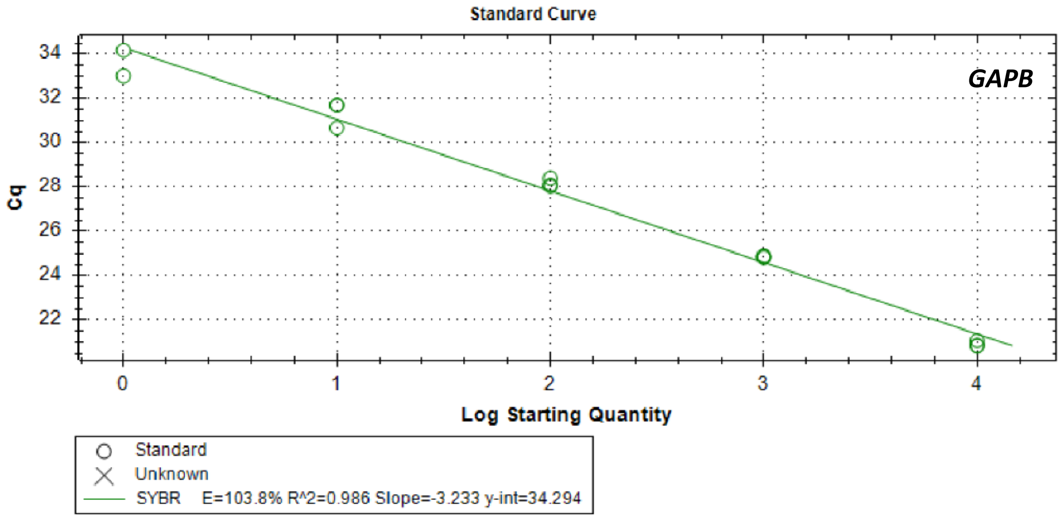
Standard Curve
GAPB
34
32
30
28
26
24
22
Cq
0
1
2
3
4
Log Starting Quantity
Standard
Unknown
SYBR E=103.8% R^2=0.986 Slope=-3.233 y-int=34.294

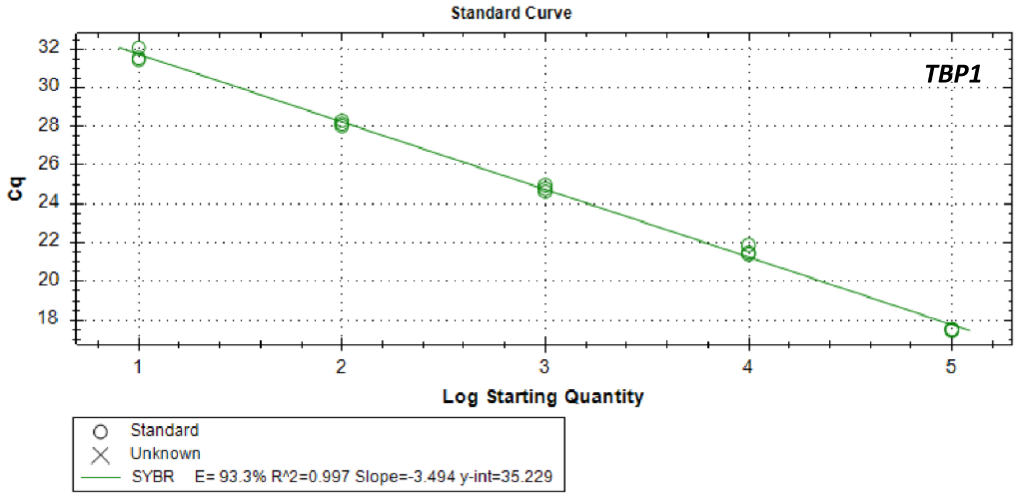
Standard Curve
TBP1
32
30
28
26
24
22
20
18
Cq
1
2
3
4
5
Log Starting Quantity
Standard
Unknown
SYBR E= 93.3% R^2=0.997 Slope=-3.494 y-int=35.229

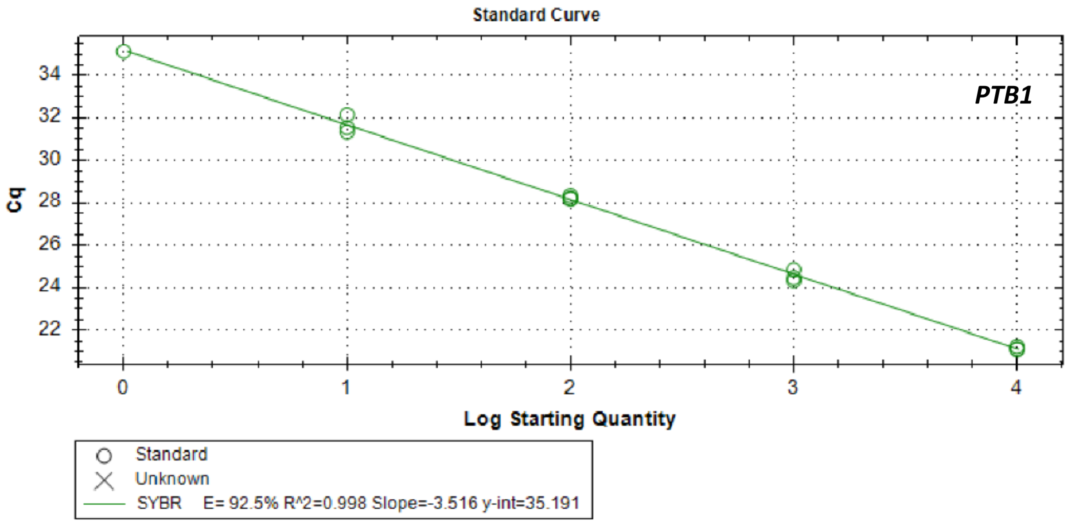

Standard Curve
PTB1
Cq
34
32
30
28
26
24
22
0
1
2
3
4
Log Starting Quantity
Standard
Unknown
SYBR E= 92.5% R^2=0.998 Slope=-3.516 y-int=35.191


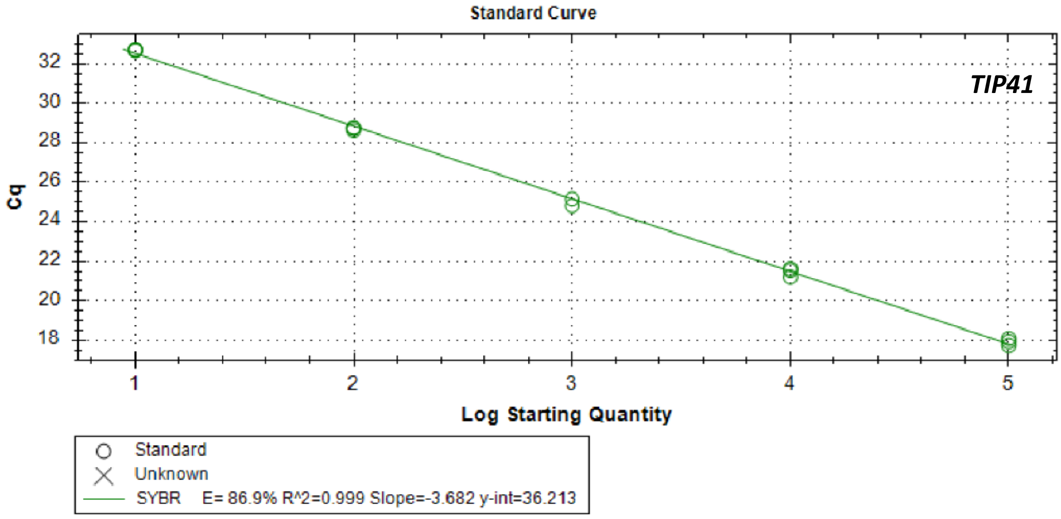

Standard Curve
TIP41
Cq
32
30
28
26
24
22
20
18
1
2
3
4
5
Log Starting Quantity
Standard
Unknown
SYBR E= 86.9% R^2=0.999 Slope=-3.682 y-int=36.213


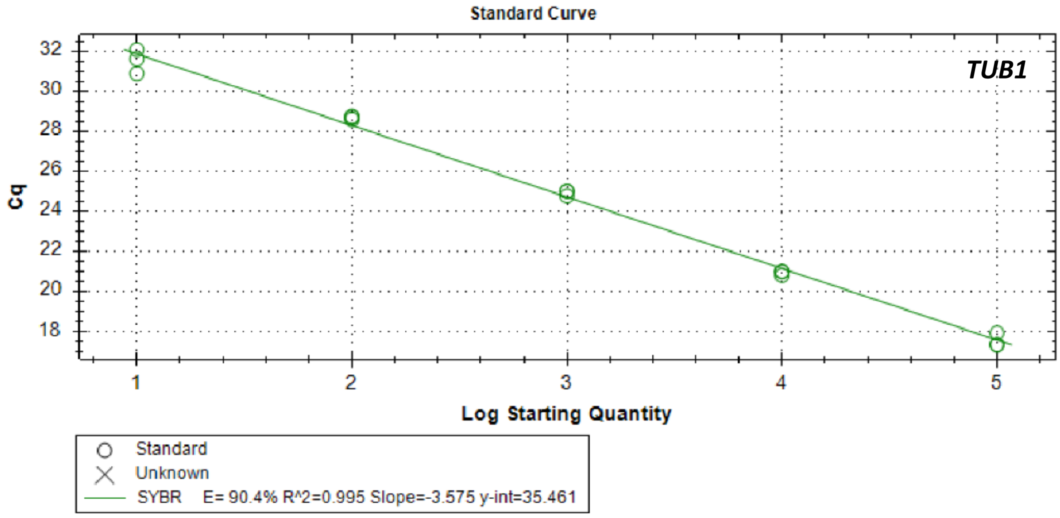

Standard Curve
TUB1
Cq
32
30
28
26
24
22
20
18
1
2
3
4
5
Log Starting Quantity
Standard
Unknown
SYBR E= 90.4% R^2=0.995 Slope=-3.575 y-int=35.461

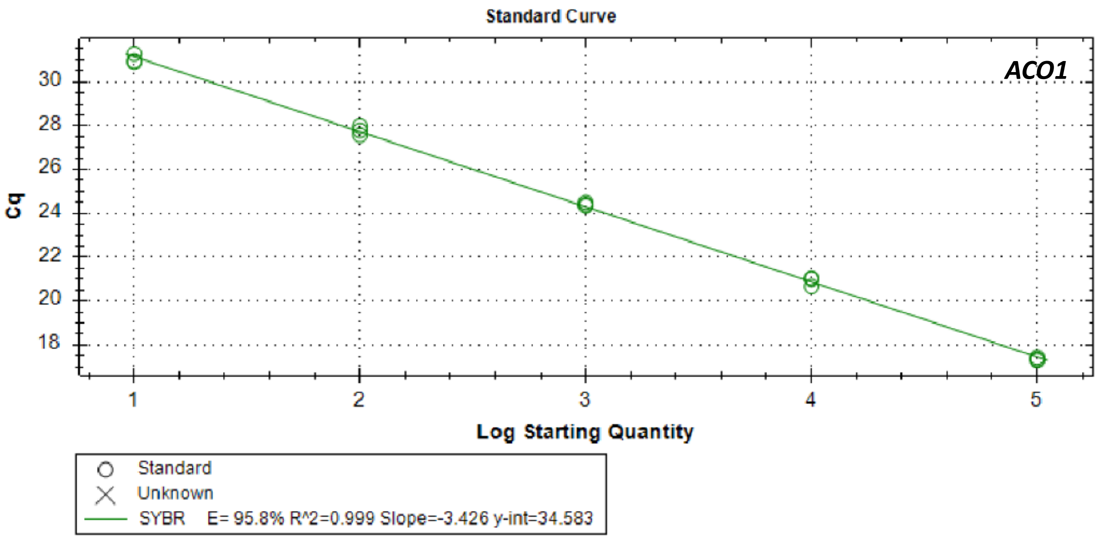
Standard Curve
ACO1
Cq
Log Starting Quantity
Standard
Unknown
SYBR E= 95.8% R^2=0.999 Slope=-3.426 y-int=34.583

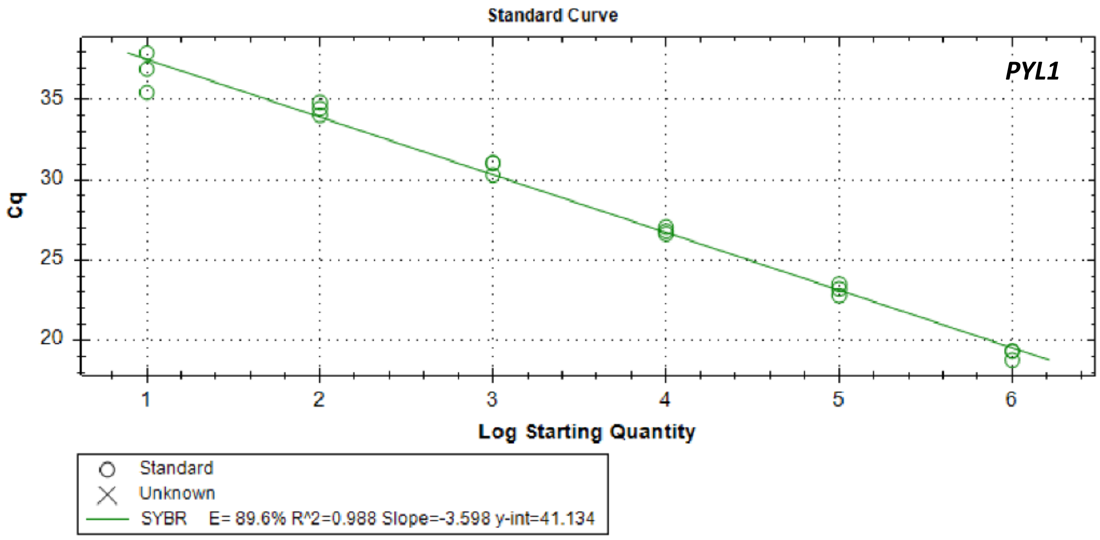
Standard Curve
PYL1
Cq
Log Starting Quantity
Standard
Unknown
SYBR E= 89.6% R^2=0.988 Slope=-3.598 y-int=41.134

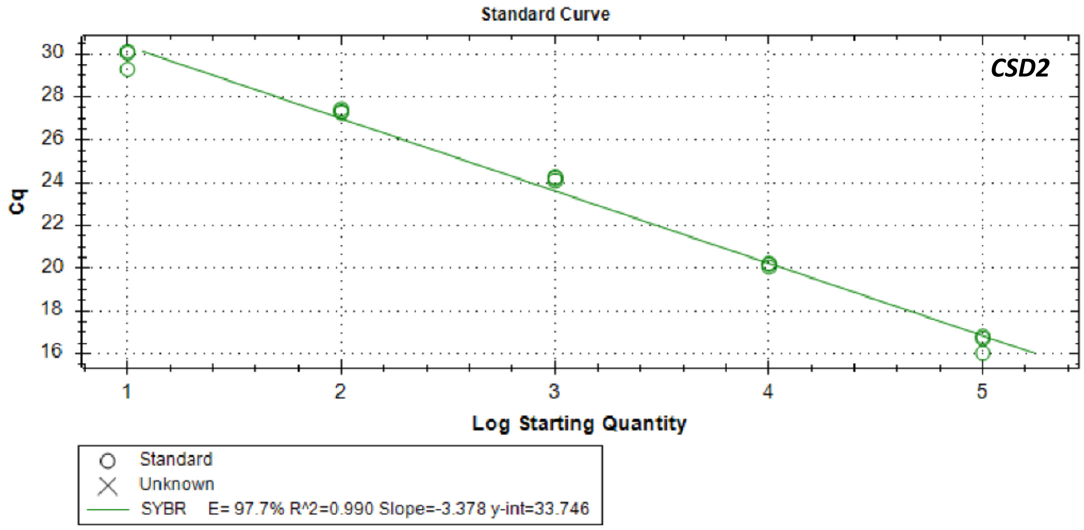
Standard Curve
CSD2
Cq
Log Starting Quantity
Standard
Unknown
SYBR E= 97.7% R^2=0.990 Slope=-3.378 y-int=33.746

**Figure S2.** Standard curves of the candidate reference genes and target genes. PCR amplification efficiency (E) and correlation coefficient ($R^2$) of primer pairs were calculated by a standard curve with a 10-fold serial dilution of the first-strand cDNA (0.05 μg/μL).
