# Supplementary material for: Identification of Reference Genes for RT-qPCR Analysis in Gleditsia microphylla under Abiotic Stress and Hormone Treatment
Source: Genes (Basel). 2022 Jul 10;13(7):1227. doi: 10.3390/genes13071227 (PMC9315665; doi:10.3390/genes13071227)
Supplement: Supplementary file 1 [file genes-13-01227-s001.zip › Figure S3 Average expression stability M value of nine candidate reference genes calculated using GeNorm by organ.pdf]

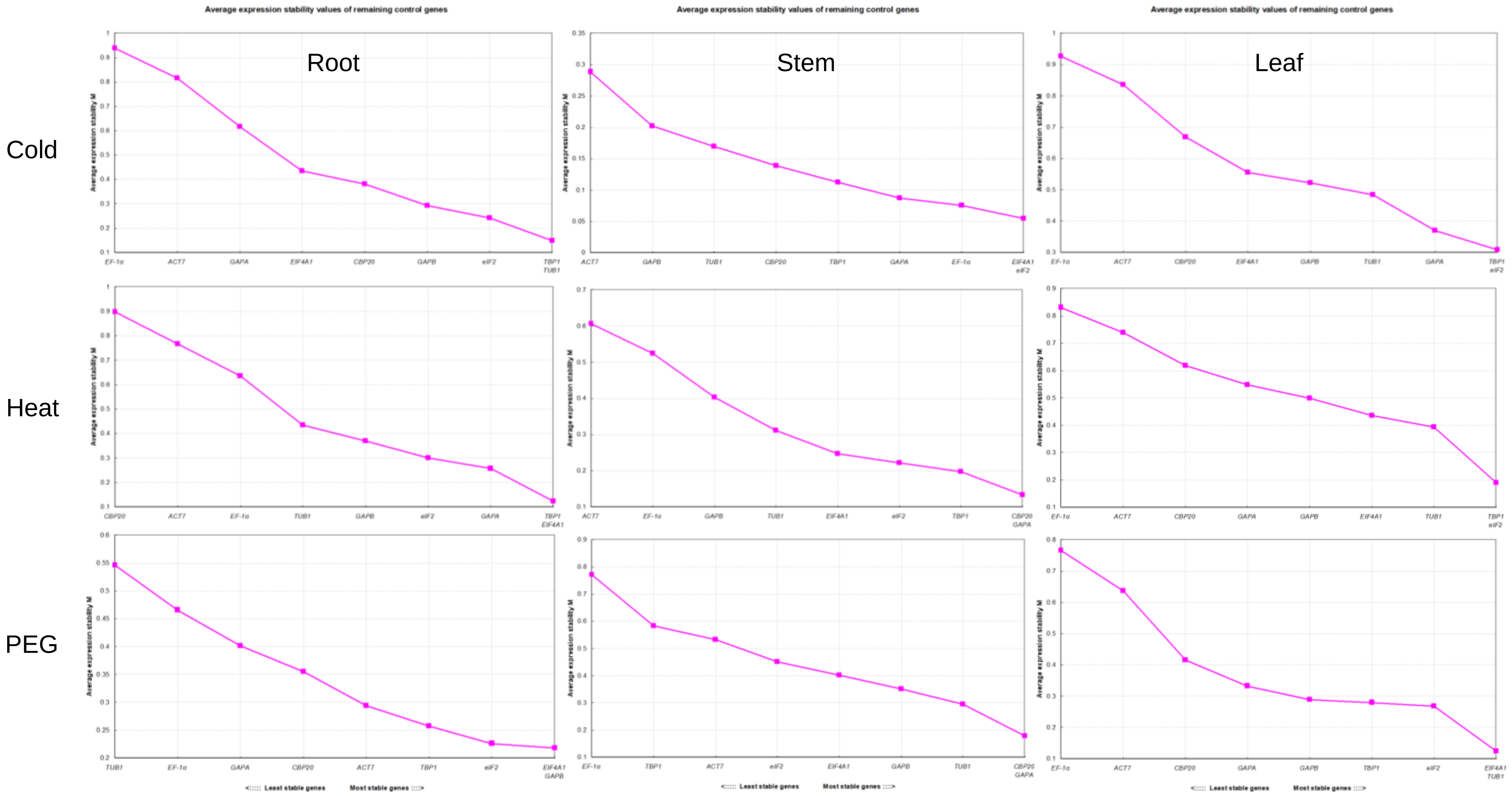

Root
Stem
Leaf
Cold
Heat
PEG
Average expression stability values of remaining control genes
Average expression stability M
<:::: Least stable genes
Most stable genes ::::>

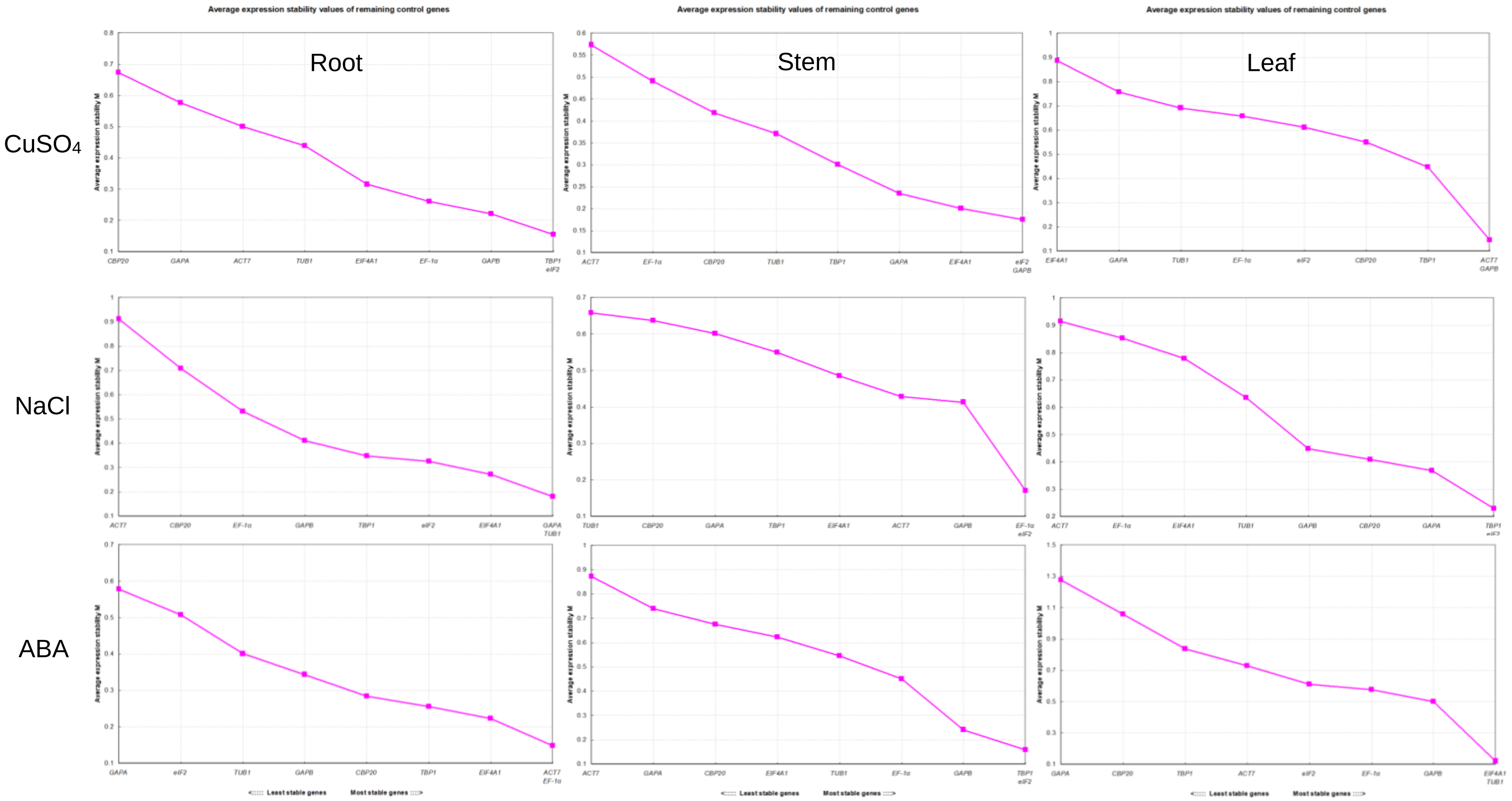
Average expression stability values of remaining control genes
Average expression stability values of remaining control genes
Average expression stability values of remaining control genes
Root
Stem
Leaf
CuSO4
NaCl
ABA
Average expression stability M
<::::: Least stable genes
Most stable genes ::::>

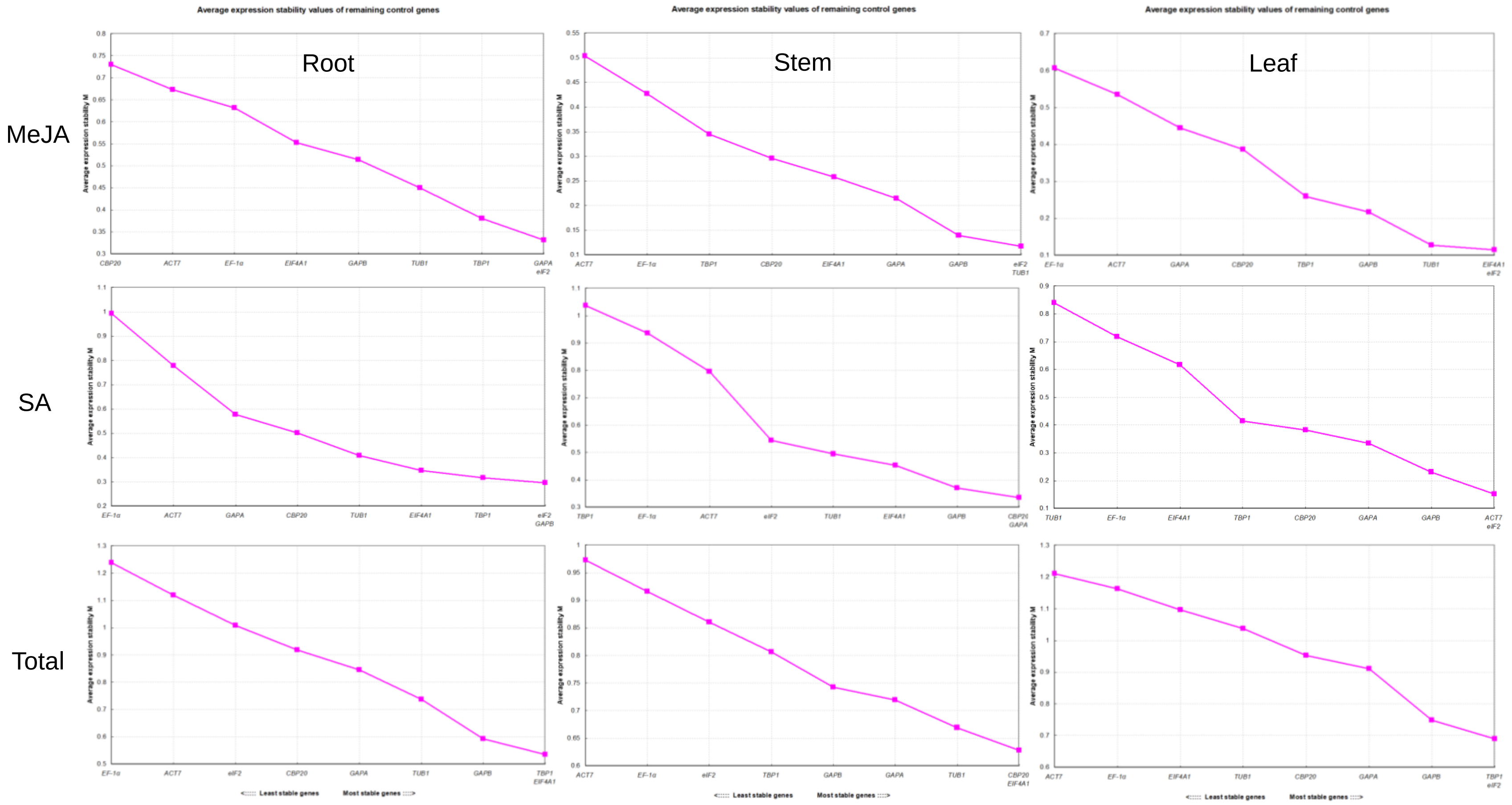

Average expression stability values of remaining control genes
Average expression stability values of remaining control genes
Average expression stability values of remaining control genes
Root
Stem
Leaf
MeJA
SA
Total
Average expression stability M
<:::: Least stable genes
Most stable genes ::::>

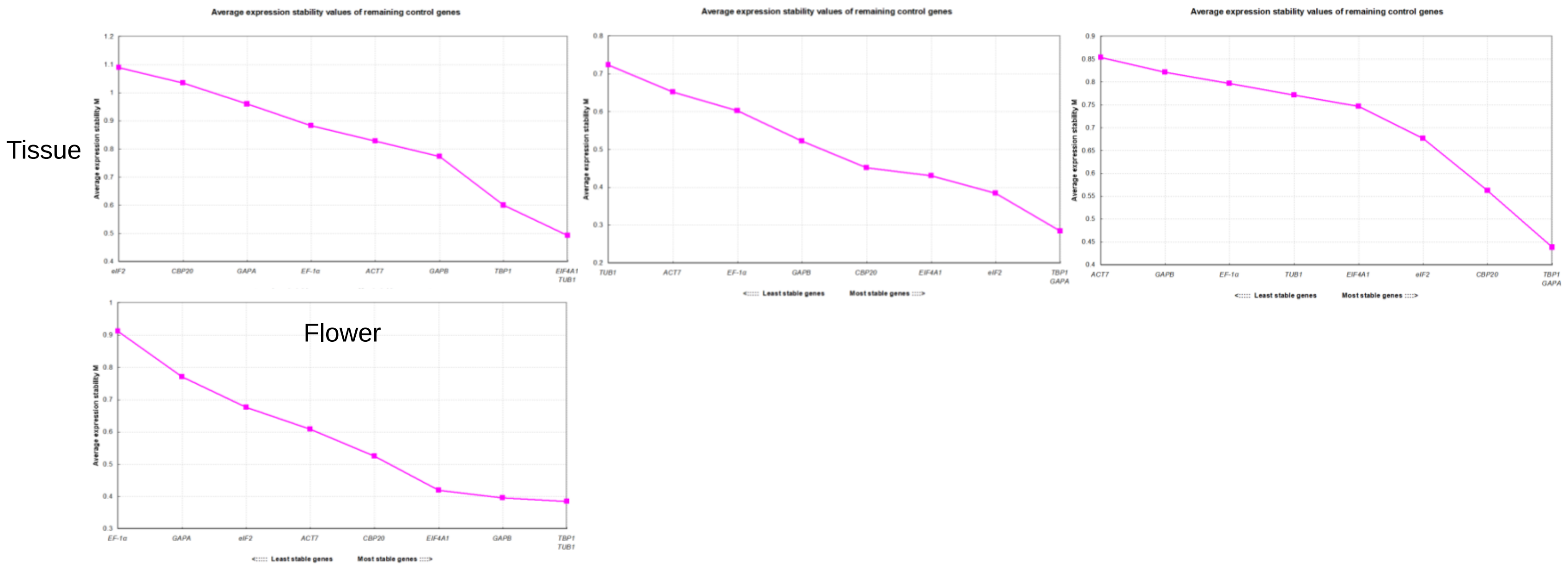

Figure S3. Average expression stability M value of nine candidate reference genes calculated using GeNorm by organ.
