# Supplementary material for: Identification of Reference Genes for RT-qPCR Analysis in Gleditsia microphylla under Abiotic Stress and Hormone Treatment
Source: Genes (Basel). 2022 Jul 10;13(7):1227. doi: 10.3390/genes13071227 (PMC9315665; doi:10.3390/genes13071227)
Supplement: Supplementary file 1 [file genes-13-01227-s001.zip › Figure S4 The pairwise variation (V) measure of the nine candidate reference genes using GeNorm by group.pdf]

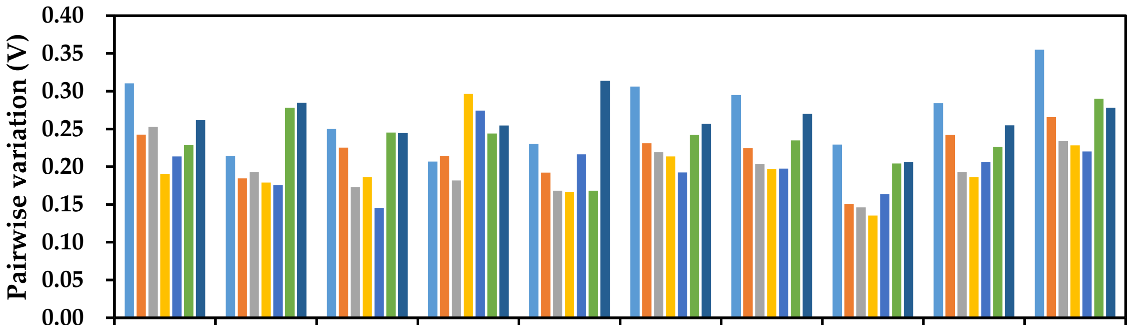

	Total	Cold	Heat	PEG	CuSO4	NaCl	ABA	MeJA	SA	Tissue
V2/3	0.310	0.214	0.250	0.207	0.230	0.306	0.295	0.229	0.284	0.355
V3/4	0.243	0.185	0.225	0.214	0.192	0.231	0.224	0.151	0.242	0.266
V4/5	0.253	0.193	0.173	0.182	0.168	0.219	0.204	0.146	0.193	0.234
V5/6	0.191	0.179	0.186	0.296	0.167	0.214	0.197	0.135	0.186	0.228
V6/7	0.214	0.176	0.146	0.274	0.216	0.192	0.197	0.164	0.206	0.220
V7/8	0.229	0.278	0.245	0.244	0.168	0.242	0.235	0.204	0.226	0.290
V8/9	0.262	0.285	0.245	0.255	0.314	0.257	0.270	0.206	0.255	0.278

Figure S4. The pairwise variation (V) measure of the nine candidate reference genes using GeNorm by group. Vn/Vn+1 values were used to calculate the optimal number of reference genes (n).
