# Supplementary material for: Identification of Reference Genes for RT-qPCR Analysis in Gleditsia microphylla under Abiotic Stress and Hormone Treatment
Source: Genes (Basel). 2022 Jul 10;13(7):1227. doi: 10.3390/genes13071227 (PMC9315665; doi:10.3390/genes13071227)
Supplement: Supplementary file 1 [file genes-13-01227-s001.zip › Table S1 Information of candidate reference genes, target genes and primers for RT-qPCR.pdf]

Table S1. Information of candidate reference genes, target genes and primers for RT-qPCR.

Gene name[a]	Description	Primer Sequence (5'-3') (For./Rev.)	Amplicon length (bp)/Tm (℃)	PCR efficiency (E)	Correlation coefficient (R^2)	GenBank Accession[b]
TBP1	TATA binding protein 1	CGGAGTACAATCCCAAGCGT/ AGCATACTTCCTTGCTGCCAA	146/77.5	93.30	0.997	MZ210062
CBP20	Nuclear cap binding protein 20	AGCTATGCCACCGTCCTATG/ ACCACTCTCCCGAAATCGC	208/78.5	118.60	0.982	MZ210063, MZ210064
TIP41	TIP41-like family protein	TCTTCTCTTGCGATTCTGGCTT/ GGTCCTCTGTATGACGATGGG	229/79.5	86.90	0.999	MZ210065, MZ210066
EF-1α	Translation elongation factor EF1A	ACAAGATGGACGCCACTACC/ AGAGGCTTGTCTGAGGGTCT	244/80.0	94.20	0.997	MZ210067, MZ210068
EIF4A1	Eukaryotic translation initiation factor 4A1	GCAAGGTGGACTGGCTTACT/ TTTCAGGTTGGGTTGGCAGA	216/79.5	92.90	0.999	MZ210069
PTB1	Polypyrimidine tract-binding protein 1	GTCAAACCTATGCGGCTCCT/ AAGGCGAAGACCGAACCAAT	240/81.5	92.50	0.998	MZ210070
ACT7	Actin 7	TTGCTGGCCGTGATCTAACT/ ACTTCTGGGCAACGGAATCT	241/79.0	94.80	1.000	MZ210071
ACT1	Actin 1	ACCCAAAGGCTAACCGTGAA/ CGTACGGCCACTAGCATACA	113/76.5	87.90	0.998	MZ210072
GAPA	Glyceraldehyde-3-phosphate dehydrogenase A subunit	GCAGAAGAAGTGAACGCTGC/ GCTGTAACCCCACTCGTTGT	201/80.5	106.50	0.990	MZ210073
GAPB	Glyceraldehyde-3-phosphate dehydrogenase B subunit	TGTTCCACTCGTGTCTGTGG/ CTGTAGCCCCACTCGTTGTC	129/78.0	103.80	0.986	MZ210074
TUB1	Tubulin beta-1 chain	CCAAGGAGGTTGACGAGCAA/ GCTCACCCTTCGGAACATCT	179/77.5	90.40	0.995	MZ210076
eIF2	Eukaryotic translation initiation factor 2	TCCTTACACTCGAACCACGC/ ACACGGACCCATTCAACTCC	149/81.0	101.90	0.998	OL456245
ACO1	1-aminocyclopropane-1-carboxylate oxidase 1	CTTGGGGACCAACTTGAGGTTATT/ ATCGTTGCCTGGGTTGTAGAAG	120/77.0	95.80	0.999	OL456246
PYL1	PYR1-like 1	CAGTATAATTGGGGGCGAGCA/ ACGATTCCAGAACCACGCTC	104/81.0	89.60	0.988	OL456247
CSD2	Copper/zinc superoxide dismutase 2	TGTTGGTGATGACGGAACTGT/ ATGCCCACCTTTCCCAAGAT	130/77.5	97.70	0.990	OL456248, OL456249

[a] Gene name based on similarity to *Arabidopsis* proteins. [b] Full-length cDNA accession number.
