# Supplementary material for: Identification of Reference Genes for RT-qPCR Analysis in Gleditsia microphylla under Abiotic Stress and Hormone Treatment
Source: Genes (Basel). 2022 Jul 10;13(7):1227. doi: 10.3390/genes13071227 (PMC9315665; doi:10.3390/genes13071227)
Supplement: Supplementary file 1 [file genes-13-01227-s001.zip › Table S2 Ct values for nine candidate reference genes under different abiotic stresses or hormone treatments.pdf]

Table S2. Ct values of nine candidate reference genes in organs under different abiotic stresses or hormone treatments.

Cold stress (4℃)

	TBP1	*ACT7*	*CBP20*	*EF-1α*	*EIF4A1*	*GAPA*	*eIF2*	*GAPB*	*TUB1*
**Root**	32.06	31.29	32.86	32.23	31.53	30.27	28.42	32.35	30.34
	32.13	31.37	32.90	32.12	31.39	30.22	28.71	32.79	30.54
	31.45	28.59	32.91	29.80	31.62	29.70	27.39	31.34	29.42
	31.28	28.88	32.89	29.75	31.65	29.95	27.56	31.29	30.01
	31.80	28.68	32.91	29.30	32.43	32.37	28.62	32.50	30.12
	32.08	28.73	32.88	29.17	32.25	32.53	28.57	32.92	30.32
**Stem**	29.18	28.17	30.84	29.33	29.50	24.21	24.62	26.95	28.91
	29.15	27.85	30.93	29.43	29.50	24.10	24.73	27.00	29.01
	29.52	27.26	30.96	29.80	29.94	24.71	25.10	26.88	29.17
	29.57	27.32	30.95	29.90	29.83	24.76	25.06	26.86	29.32
	29.56	26.75	30.94	29.50	29.80	24.28	24.85	26.40	28.45
	29.06	26.91	30.94	29.50	29.63	24.33	24.91	26.55	28.93
**Leaf**	28.16	28.46	29.89	30.94	30.12	22.58	24.40	25.50	29.41
	28.10	28.98	29.80	30.84	30.24	22.28	24.48	25.41	29.82
	29.32	28.22	30.27	31.20	30.58	22.62	25.04	25.40	29.90
	29.40	28.21	30.36	31.28	30.70	22.61	25.06	25.37	30.36
	28.18	25.14	30.57	27.60	28.30	22.18	24.12	23.76	28.07
	28.22	25.22	30.88	27.53	28.39	22.20	23.84	23.89	28.54

Heat stress (42℃)

	TBP1	*ACT7*	*CBP20*	*EF-1α*	*EIF4A1*	*GAPA*	*eIF2*	*GAPB*	*TUB1*
**Root**	31.76	32.40	31.94	32.22	31.11	30.67	30.60	32.05	32.22
	31.66	32.36	31.67	31.82	31.25	30.60	30.53	31.96	32.33
	31.02	31.09	30.91	31.83	30.57	30.03	29.87	31.43	30.83
	31.03	30.89	31.00	31.67	30.80	29.86	30.01	31.34	30.91
	31.47	28.58	32.64	29.16	31.01	29.66	29.41	30.41	30.09
	31.36	28.71	32.89	29.05	30.79	29.73	29.40	30.44	30.50
**Stem**	30.12	30.25	30.14	30.16	29.18	24.07	27.41	27.44	28.53
	29.65	30.20	30.10	30.04	29.09	24.04	27.28	27.39	28.72
	29.53	28.85	29.70	29.33	29.25	23.86	26.85	26.25	29.18
	29.38	28.61	29.65	29.28	29.22	23.86	26.93	26.15	29.28
	29.88	27.90	30.19	28.21	29.66	24.14	26.97	26.25	28.76
	29.55	28.11	30.43	28.13	29.57	24.33	26.97	26.27	29.14
**Leaf**	28.26	29.91	28.83	29.08	29.75	22.51	26.01	25.17	30.12
	28.22	29.94	28.80	30.58	29.86	22.55	26.03	25.19	30.35
	29.13	29.47	30.46	30.26	31.13	22.61	27.29	25.04	30.54
	29.09	29.30	30.56	30.35	31.09	22.64	27.19	24.78	30.66
	28.69	27.74	30.29	27.66	29.60	22.94	26.39	24.40	29.44
	28.48	27.54	30.30	27.71	29.17	22.96	26.39	24.20	29.63

Drought stress (PEG)

	TBP1	*ACT7*	*CBP20*	*EF-1α*	*EIF4A1*	*GAPA*	*eIF2*	*GAPB*	*TUB1*
**Root**	31.94	29.71	32.43	28.45	31.51	30.83	32.25	32.26	31.25
	31.53	29.80	31.69	27.24	31.40	30.55	32.10	32.11	31.33
	31.47	29.05	32.44	28.13	31.21	30.11	31.46	31.72	30.26
	30.73	28.96	32.39	28.16	31.17	30.11	31.45	31.89	30.33
	31.46	28.79	32.24	27.38	31.78	31.23	32.21	31.95	32.17
	31.33	29.06	32.35	27.20	31.25	31.36	31.94	31.95	32.34
**Stem**	30.74	29.04	32.25	28.71	30.80	26.22	28.12	27.93	29.69
	30.61	29.01	32.29	28.54	30.71	26.16	28.09	28.07	29.95
	30.19	28.15	31.09	29.06	29.47	24.75	26.80	26.47	28.93
	29.99	28.20	30.54	28.83	29.58	24.81	26.60	26.40	29.19
	29.25	27.11	28.08	28.61	28.09	21.84	25.60	24.50	26.23
	29.11	27.48	28.70	29.24	27.92	22.66	25.58	24.94	26.82
**Leaf**	29.22	30.00	29.82	30.10	30.40	23.35	26.37	25.77	30.19
	29.30	29.71	29.44	30.19	30.44	23.40	26.42	25.70	30.30
	29.49	27.58	30.17	27.60	29.68	23.98	26.44	25.41	29.38
	29.41	27.63	30.39	27.54	30.05	23.99	26.37	25.37	29.99
	29.16	27.24	30.82	27.36	29.96	23.42	26.29	25.00	29.73
	29.34	26.93	30.53	27.43	29.67	23.46	26.68	25.02	29.90

Heavy metal stress ($CuSO_4$)

	TBP1	*ACT7*	*CBP20*	*EF-1α*	*EIF4A1*	*GAPA*	*eIF2*	*GAPB*	*TUB1*
**Root**	31.89	32.96	30.23	32.26	31.19	30.90	30.73	31.54	32.86
	32.14	32.90	30.34	32.23	31.50	31.70	30.82	31.37	32.70
	32.06	32.72	30.29	32.37	31.28	31.85	31.12	31.35	32.91
	32.70	32.06	32.58	32.95	32.08	31.01	31.56	31.94	32.05
	32.69	32.17	32.64	32.65	32.37	30.71	31.47	31.90	31.88
	32.50	32.00	32.47	32.05	32.05	31.05	31.91	31.46	31.80
	32.65	32.10	32.31	32.56	32.79	32.97	31.85	31.24	32.55
	32.43	32.08	31.57	32.59	32.56	32.83	31.95	32.08	32.66
	33.00	32.22	32.48	32.49	32.84	32.44	31.54	32.25	32.56
**Stem**	30.55	30.40	30.46	31.36	30.45	24.27	26.78	27.88	30.55
	30.40	30.26	30.44	31.32	30.17	24.21	26.68	27.68	30.62
	30.58	30.58	30.50	31.42	30.62	24.26	26.77	27.74	30.85
	30.55	28.69	30.99	30.33	30.61	24.36	27.10	27.71	30.22
	30.55	28.73	31.20	30.43	31.17	24.43	27.14	27.78	30.29
	30.55	28.71	31.08	30.38	30.98	24.38	27.06	27.86	30.30
	31.29	29.28	31.88	30.46	30.61	24.87	27.06	27.70	31.54
	31.45	29.32	32.04	30.63	30.70	24.93	27.10	27.76	31.58
	31.61	29.57	32.35	30.67	30.73	24.69	27.10	27.88	31.76
**Leaf**	29.82	30.03	30.38	30.77	29.72	23.11	25.63	26.90	30.42
	29.64	29.75	30.07	30.60	29.67	23.51	25.65	26.64	31.46
	29.70	30.00	30.14	30.68	29.71	23.28	25.68	27.04	31.49
	30.39	29.65	31.52	31.55	32.51	22.27	27.32	26.34	32.54
	30.16	29.62	31.45	31.44	32.41	22.32	27.37	26.41	32.66
	30.29	29.62	31.51	31.48	32.44	22.31	27.54	26.36	32.81
	30.45	30.52	31.73	30.70	30.04	24.33	27.34	27.31	31.61
	32.04	30.83	32.12	30.88	30.09	24.53	27.34	27.21	31.68
	31.29	30.54	31.81	30.81	30.08	24.22	27.40	27.33	31.78

Salinity stress (NaCl)

	TBP1	*ACT7*	*CBP20*	*EF-1α*	*EIF4A1*	*GAPA*	*eIF2*	*GAPB*	*TUB1*
	32.32	32.84	30.51	31.40	32.32	30.18	30.55	32.85	32.78
	31.96	32.07	30.48	31.34	32.27	29.82	30.48	32.87	32.12
	31.96	32.25	30.33	31.57	32.11	29.66	30.44	32.92	32.32
	32.86	32.93	32.33	30.80	32.70	30.34	31.34	32.76	32.51
**Root**	32.67	32.76	32.21	31.41	32.58	29.75	31.67	32.65	32.38
	33.00	32.83	32.38	31.25	32.95	30.18	31.33	32.70	32.31
	31.27	28.29	31.81	28.74	31.14	29.03	30.04	31.00	31.24
	31.50	28.60	32.34	28.78	31.49	29.53	29.72	31.18	31.69
	32.61	28.62	32.45	29.00	31.56	29.70	29.85	31.37	31.95
	30.35	29.99	30.14	30.98	30.23	24.71	27.50	28.16	29.10
	30.14	29.91	30.06	30.71	30.19	24.62	27.43	28.13	29.23
	30.61	30.04	30.15	31.03	30.38	24.94	27.58	28.31	29.29
	31.23	30.33	31.69	32.46	32.25	24.61	29.06	28.46	30.43
**Stem**	31.51	30.34	31.71	32.53	32.79	24.63	28.97	28.45	30.50
	31.19	30.28	31.63	32.05	32.16	24.70	29.06	28.56	30.52
	30.69	28.61	31.18	30.29	30.11	24.43	27.10	27.23	30.05
	30.65	28.34	30.61	30.15	29.98	24.21	27.10	27.27	30.33
	30.81	28.68	31.33	30.32	30.20	24.53	27.18	27.15	30.43
	30.17	31.97	30.15	31.38	31.76	22.59	27.59	27.05	32.01
	29.94	31.71	29.58	31.48	31.58	21.96	27.38	26.94	32.30
	30.08	31.86	29.76	31.25	31.72	22.57	27.34	27.12	32.36
	30.30	30.36	29.93	32.83	31.62	22.80	27.47	26.41	32.42
**Leaf**	30.77	30.59	30.07	32.93	31.78	23.08	27.55	26.50	32.45
	30.66	30.50	29.94	32.88	31.74	22.96	27.55	26.44	32.59
	30.39	29.84	31.02	30.47	29.64	23.44	27.39	26.87	30.35
	30.40	29.87	31.03	30.59	29.97	23.59	27.43	26.70	30.74
	30.38	28.60	30.71	29.89	28.96	23.18	27.34	26.73	31.22

ABA treatment

	TBP1	*ACT7*	*CBP20*	*EF-1α*	*EIF4A1*	*GAPA*	*eIF2*	*GAPB*	*TUB1*
**Root**	32.40	31.39	31.60	32.35	31.23	30.15	31.22	32.50	30.44
	32.18	31.36	31.61	31.98	31.24	29.91	31.19	32.59	30.65
	32.32	31.22	31.73	32.40	31.45	29.86	31.23	32.41	30.87
	32.06	30.52	31.55	31.73	31.01	29.11	30.75	31.13	30.58
	31.83	30.25	31.34	31.43	31.00	28.76	30.40	31.27	30.92
	32.26	30.62	31.55	31.61	31.03	29.16	30.59	31.43	31.00
	32.28	31.51	31.60	32.34	31.41	31.78	32.94	32.83	30.30
	32.09	31.57	31.53	32.39	31.89	31.65	32.72	32.84	30.38
	32.68	31.44	31.37	32.33	31.82	31.20	32.78	32.80	30.40
**Stem**	30.82	30.02	30.68	30.83	30.05	25.10	27.54	27.83	29.95
	30.67	29.91	30.69	30.72	30.00	25.01	27.39	27.79	30.02
	30.69	30.08	30.82	31.36	30.16	25.12	27.64	27.90	30.05
	31.67	29.86	31.34	30.91	30.35	26.01	28.10	28.69	29.01
	31.54	29.72	31.22	30.48	30.26	25.95	28.02	28.59	29.05
	31.39	29.87	31.38	30.85	30.16	25.88	28.10	28.62	29.26
	30.92	31.10	28.57	29.46	27.84	22.68	27.39	27.39	28.29
	30.59	30.80	28.40	29.38	27.90	22.32	27.45	27.32	28.36
	30.95	31.05	28.48	29.29	27.95	22.58	27.43	27.40	28.38
**Leaf**	30.30	32.92	28.37	29.86	30.47	21.08	27.62	26.19	31.38
	30.58	32.33	28.37	29.96	30.27	21.50	27.70	26.34	31.43
	30.65	32.57	28.38	29.01	30.23	21.72	27.53	26.29	31.60
	30.94	30.00	30.64	29.74	29.00	23.18	27.14	26.02	29.96
	30.68	30.07	30.74	29.73	29.03	23.17	26.90	26.00	29.98
	30.81	30.05	30.72	29.63	29.04	23.32	27.15	26.19	30.05
	31.97	30.09	31.78	29.90	28.83	24.07	27.90	26.18	29.89
	32.23	30.35	31.64	29.82	28.90	24.26	28.35	26.20	29.91
	32.33	30.32	31.08	29.70	28.56	27.76	28.01	26.26	29.95

MeJA treatment

	TBP1	*ACT7*	*CBP20*	*EF-1α*	*EIF4A1*	*GAPA*	*eIF2*	*GAPB*	*TUB1*
	31.32	30.53	30.64	30.89	31.14	28.30	29.86	31.48	30.78
	31.27	30.93	31.06	30.55	31.10	28.90	30.16	31.58	31.07
	31.72	30.98	31.15	31.14	31.73	28.88	30.00	31.45	31.16
	32.82	30.00	32.91	30.39	31.65	29.87	30.99	31.92	31.13
**Root**	32.72	30.10	32.71	30.44	31.15	29.81	30.86	32.03	31.14
	32.85	30.15	32.37	29.85	30.84	29.95	30.94	31.87	31.31
	32.66	29.15	31.23	28.96	30.85	30.63	30.56	30.51	30.97
	32.65	30.53	31.38	30.98	32.87	30.22	31.95	32.20	31.73
	32.64	30.23	31.24	30.89	32.65	30.44	31.60	32.37	32.08
	29.80	28.66	29.23	28.07	29.25	24.49	27.00	26.75	28.89
	29.96	28.84	30.22	29.04	29.57	24.57	27.30	27.34	29.23
	29.84	28.90	30.19	29.05	29.63	24.40	27.28	27.31	29.23
	30.92	28.91	30.46	30.11	29.49	24.59	27.41	27.53	29.24
**Stem**	31.16	28.98	30.58	30.23	29.78	24.67	27.46	27.61	29.29
	31.17	28.64	30.34	29.72	29.58	24.71	27.45	27.50	29.38
	31.28	28.35	31.64	29.44	31.20	25.50	27.87	28.08	29.78
	31.33	28.25	31.29	29.36	30.68	25.86	27.83	28.11	29.99
	31.61	28.31	31.70	29.38	30.88	25.88	27.80	28.11	30.00
	29.60	29.28	28.78	29.62	29.94	21.86	26.59	25.63	30.14
	29.49	29.20	28.75	29.62	29.86	21.97	26.56	25.61	30.20
	29.99	29.36	28.79	29.76	30.06	22.16	26.58	25.67	30.37
	30.61	28.61	30.42	29.02	29.98	23.70	26.57	26.23	30.17
**Leaf**	30.32	28.44	30.44	29.11	30.08	23.80	26.40	26.14	30.31
	30.24	28.55	30.39	29.06	29.92	23.80	26.36	26.15	30.39
	30.53	28.38	30.32	28.53	30.24	23.61	26.97	26.34	30.42
	30.50	28.36	30.23	28.17	30.39	23.17	26.98	25.69	30.51
	30.48	28.24	29.98	28.22	30.57	23.65	26.93	25.97	30.55

SA treatment

	TBP1	*ACT7*	*CBP20*	*EF-1α*	*EIF4A1*	*GAPA*	*eIF2*	*GAPB*	*TUB1*
	31.32	31.33	29.95	32.84	30.57	28.47	30.64	31.46	29.83
	31.07	31.22	29.96	32.75	30.56	28.17	30.53	31.51	29.87
	31.30	31.30	29.93	32.77	30.37	28.65	30.66	31.66	29.99
	32.58	30.15	32.46	30.27	32.38	31.55	32.02	32.34	31.56
**Root**	32.87	30.07	32.59	30.25	32.33	31.76	32.19	32.68	31.68
	32.92	30.22	32.84	30.80	32.78	32.09	32.13	32.45	31.85
	31.78	31.32	32.55	32.78	31.63	30.57	31.72	32.15	31.80
	31.81	31.37	32.68	32.69	32.20	30.98	32.08	32.28	31.96
	31.93	31.72	32.58	32.58	32.00	30.95	31.70	32.15	32.00
	31.82	31.46	30.59	32.60	31.26	25.13	27.67	28.43	30.70
	31.79	31.68	30.54	32.71	31.22	25.04	27.71	28.35	30.74
	31.69	31.53	30.62	32.52	31.40	25.13	27.80	28.38	30.81
	31.68	29.41	30.85	30.68	30.43	25.59	27.60	28.09	29.99
**Stem**	31.63	29.52	30.79	30.84	30.43	25.48	27.65	28.07	30.11
	31.70	29.29	30.90	30.78	30.69	25.57	27.62	27.93	30.12
	30.76	30.53	32.91	31.38	32.79	28.14	30.80	30.88	31.61
	31.04	30.61	32.85	31.39	32.71	28.32	31.39	30.92	32.00
	31.32	30.73	32.81	31.36	32.96	27.70	31.02	31.05	32.05
	29.67	29.96	29.19	31.32	30.63	22.45	27.18	25.72	30.60
	29.26	29.81	29.06	31.35	30.79	22.13	27.39	25.60	30.91
	29.61	29.90	29.18	31.36	30.75	22.44	27.26	25.75	30.93
	30.42	29.72	29.95	29.66	28.86	22.63	27.31	26.07	30.80
**Leaf**	30.69	29.83	29.97	29.81	28.95	22.79	27.49	26.02	30.98
	30.60	29.75	29.90	29.52	28.91	22.68	27.61	26.04	31.14
	32.06	30.64	31.37	30.39	30.29	24.21	28.11	27.01	29.45
	31.90	30.63	31.44	30.45	30.03	24.25	28.08	27.05	29.65
	31.81	30.57	31.18	30.54	30.06	24.29	28.07	27.09	29.77

Tissues

	TBP1	*ACT7*	*CBP20*	*EF-1α*	*EIF4A1*	*GAPA*	*eIF2*	*GAPB*	*TUB1*
**Root**	32.69	29.23	30.72	30.56	31.88	30.58	31.50	32.01	31.87
	32.19	29.27	30.91	30.83	31.91	30.95	31.92	32.10	32.48
	32.50	29.49	30.46	30.31	31.15	30.58	31.88	32.35	32.62
	31.63	29.69	32.02	30.25	31.97	30.03	31.02	32.23	31.88
	30.93	28.90	31.72	30.10	31.68	30.15	30.97	32.32	31.99
	31.88	29.99	32.08	30.29	32.10	29.99	31.29	32.27	32.04
	31.67	28.42	31.30	29.03	31.11	30.81	30.93	31.58	30.93
	32.45	29.40	31.33	29.79	32.17	30.61	31.66	32.72	31.70
	32.66	29.46	31.22	30.05	32.33	30.43	31.37	32.76	31.80
	30.84	29.16	31.29	28.49	29.18	29.89	31.93	32.26	29.64
	30.95	29.19	31.21	28.72	29.23	29.95	32.00	32.34	29.67
	31.21	28.59	31.92	28.21	29.77	29.44	31.33	31.85	29.05
	30.58	28.51	32.44	28.18	29.91	29.62	31.74	32.10	29.41
	30.97	27.78	32.36	26.77	30.54	32.04	31.46	32.59	29.62
	30.93	27.86	32.37	26.58	30.30	32.28	31.80	32.53	30.16
	30.86	30.33	29.62	29.53	30.63	30.80	31.56	32.35	30.03
	30.61	30.22	30.35	29.18	30.68	30.51	31.25	31.82	30.28
	31.74	29.64	31.92	28.66	31.71	31.06	27.62	32.43	31.46
	31.56	29.42	32.16	28.26	31.57	31.07	31.51	32.35	31.55
	32.04	29.16	32.80	28.47	32.00	31.37	32.13	32.23	31.28
	31.11	27.93	32.37	27.60	30.74	31.28	31.27	30.69	31.38
**Stem**	29.00	28.96	29.76	29.46	29.30	23.57	27.37	26.68	30.13
	30.10	30.18	30.66	30.42	30.33	24.01	27.80	27.51	30.59
	30.19	30.30	31.00	30.63	30.52	23.63	27.85	27.52	30.89
	30.65	29.47	30.70	30.72	29.59	24.06	27.95	27.56	29.54
	30.60	29.36	30.61	30.70	29.52	24.15	28.06	27.58	29.65
	30.06	29.31	30.18	30.53	29.51	24.13	28.05	27.58	29.77

**Stem**	31.77	29.05	32.10	30.25	31.18	26.16	29.38	28.80	30.51
	31.83	29.41	32.73	30.25	31.30	26.19	29.53	28.83	30.52
	32.06	29.49	32.94	30.27	31.33	25.97	29.28	29.00	30.68
	29.37	28.39	30.51	28.89	29.69	23.42	27.28	25.92	28.68
	29.33	28.36	30.63	28.79	29.53	23.49	27.34	25.84	28.79
	29.82	28.10	30.32	29.66	28.61	23.36	27.38	25.35	29.01
	29.78	28.12	30.36	29.66	28.58	23.34	27.20	25.05	29.01
	30.17	28.16	31.34	30.01	29.64	23.94	27.71	26.21	28.10
	30.15	28.04	31.51	29.91	29.66	24.15	27.70	26.25	28.63
	30.30	29.43	31.05	29.64	29.85	24.19	27.01	26.13	29.66
	30.91	30.09	31.09	30.41	30.38	24.85	27.24	26.88	30.06
	30.33	28.97	30.25	30.27	29.70	24.39	27.67	27.06	28.64
	30.48	29.16	30.25	30.20	29.62	24.45	27.62	27.05	28.70
	30.80	29.03	30.74	31.06	29.95	24.59	27.93	27.48	27.69
	30.67	28.99	30.93	31.10	29.84	24.51	27.91	27.60	27.71
**Leaf**	29.46	30.68	30.82	31.32	30.16	22.14	28.02	26.33	29.38
	27.99	29.59	30.61	29.39	28.86	22.30	27.93	26.32	30.99
	28.93	30.27	30.74	30.69	29.69	21.97	27.87	26.27	31.38
	29.63	28.58	29.92	29.45	28.25	22.03	27.69	25.37	29.37
	29.19	28.50	29.89	28.97	28.14	21.88	27.75	25.33	29.50
	29.64	28.72	30.03	29.52	28.34	22.07	27.64	25.32	29.74
	28.51	26.68	30.29	28.03	28.25	21.70	27.14	24.40	28.34
	28.72	26.78	30.32	28.28	28.37	21.82	27.39	24.47	29.10
	28.45	26.66	30.05	27.53	27.59	21.71	27.40	24.55	29.25
	30.08	30.02	30.66	31.02	29.79	22.69	27.74	25.02	30.29
	29.89	29.96	30.90	30.87	29.61	22.82	27.80	25.16	30.93
	29.77	27.92	30.47	29.90	29.15	22.11	27.91	24.13	30.59
	29.75	27.91	30.11	29.93	29.12	22.12	28.05	24.08	30.70
	30.06	27.90	31.95	29.07	28.81	23.06	27.40	24.17	29.23
	29.96	27.82	31.06	29.09	28.81	23.09	27.48	24.28	29.35

	29.34	29.60	29.36	30.28	30.13	22.47	26.61	24.69	29.83
	29.33	28.80	29.66	29.59	30.30	22.54	26.76	24.91	30.46
	29.16	28.13	29.70	28.83	28.89	22.58	26.49	24.22	29.19
	29.20	28.12	29.67	28.62	28.88	22.59	26.54	24.21	29.35
	29.69	27.14	30.67	28.18	28.40	23.15	26.26	23.82	28.33
	29.45	27.41	30.54	27.84	28.29	23.10	26.42	24.22	28.64
**Flower**	29.77	29.05	27.22	31.58	27.60	22.34	29.08	26.17	26.31
	29.70	28.92	27.30	31.59	27.57	22.53	28.99	26.32	26.32
	29.27	28.22	26.06	28.92	27.36	23.71	26.93	26.26	26.49
	29.31	28.47	26.19	29.08	27.27	23.63	26.92	26.22	26.76
	28.47	27.48	25.96	29.72	26.40	22.04	26.10	25.23	24.97
	28.46	27.48	25.93	29.69	26.38	22.17	26.04	25.20	24.98
	29.98	27.95	27.32	29.42	28.21	23.71	27.45	26.73	27.49
	30.25	28.02	27.26	29.44	28.07	23.73	27.48	26.75	27.51
	29.25	28.17	26.92	29.38	27.95	25.02	26.98	26.86	26.45
	29.34	28.29	27.35	29.31	28.07	24.81	26.78	26.90	26.45
	28.58	26.10	27.00	26.26	25.64	23.37	25.69	25.16	25.28
	28.72	26.04	26.91	26.39	25.67	23.36	25.69	25.24	25.37
